# Impact of food texture modifications on oral processing behaviour, bolus properties and postprandial glucose responses

**DOI:** 10.1016/j.crfs.2021.11.018

**Published:** 2021-12-01

**Authors:** J.Y.M. Choy, A.T. Goh, G. Chatonidi, S. Ponnalagu, S.M.M. Wee, M. Stieger, C.G. Forde

**Affiliations:** a Clinical Nutrition Research Centre (CNRC), Singapore Institute for Food and Biotechnology Innovation), Agency for Science, Technology and Research (A*STAR), Singapore; b Wageningen University, Sensory Science and Eating Behaviour, Division of Human Nutrition and Health, P.O. Box 17, 6700, AA Wageningen, the Netherlands

**Keywords:** Food texture, Oral processing behaviour, Bolus particle size, Bolus surface area, Salivary α–amylase, Glycemic response, OPB, Oral processing behaviours, iAUC, incremental Area under the curve, PPG, Postprandial glycaemic response, SIR, Saliva incorporation rate, TPA, Texture profile analysis, VAS, Visual analogue scale

## Abstract

Several studies have demonstrated food texture manipulation on oral processing behaviour (OPB). We explored the effect of texture-differences of equivalent carbohydrate load on OPB, bolus properties and postprandial glycaemic responses (PPG). In a randomised cross-over, within-subjects, non-blinded design, healthy male participants (N = 39) consumed fixed portions of white rice (WR) and rice cake (RC) while being video recorded to measure microstructural eating behaviours. PPG was compared between test foods over a period of 120-min, and the bolus properties and saliva uptake at swallow were measured for both test foods. RC displayed higher instrumental hardness, chewiness and Young's modulus than WR (*p* = 0.01), and participants perceived RC as more *springy* and *sticky* than WR (*p* < 0.001). The RC meal was chewed more per bite (*p* < 0.001) and consumed at a faster eating rate (*p* = 0.033) than WR. WR bolus particles were smaller at swallow (*p* < 0.001) with a larger total surface area (*p* < 0.001), compared to RC. The glucose response for RC was significantly higher during the first 30-min postprandial period (p = 0.010), and lower in the later (30–120 min) postprandial period (*p* = 0.031) compared to WR. Total blood glucose iAUC did not differ significantly between WR and RC meals despite their large differences in texture, OPB and bolus properties. Oro-sensory exposure time was a significant predictor of glucose iAUC_30min_ for both test meals (RC, *p* = 0.003; WR, *p* = 0.029). Saliva uptake in the bolus was significantly positively associated with blood glucose during the first 30-min postprandial period for the RC meal (*p* = 0.008), but not for WR. We conclude that food texture modifications can influence OPB and bolus properties which are key contributors to the dynamic evolution of the glycaemic response. Total blood glucose responses were the same for both test foods, though differences in oral processing and bolus properties influenced temporal changes in PPG.

## Highlights

• Food texture differences influences oral processing, bolus particle size and saliva uptake.
• Increased bolus surface area, saliva uptake and oro-sensory exposure time increases glycaemic excursions.
• Salivary α-amylase concentration does not influence postprandial glycaemic response.

## Graphical abstract

Natural adjustments of oral processing behaviours were observed in response to food texture modification, producing large differences in eating microstructure, bolus properties and subsequent temporal postprandial glucose responses.

**Figure:**
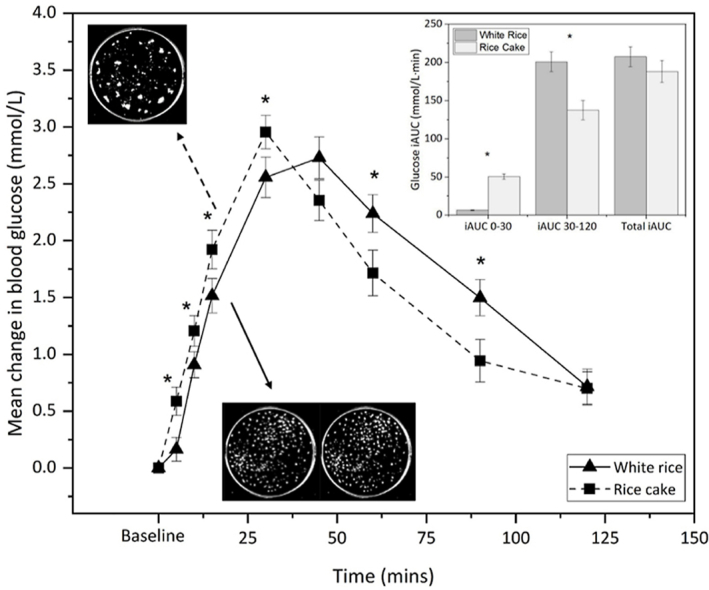

## 1 Introduction

Sustained hyperglycaemia is associated with an increased risk of developing type-2 diabetes, and it is recommended to reduce hyperglycaemic peaks and maintain blood glucose within the normal range (Khan et al., 2019). Several studies have demonstrated that starch digestion in the oral phase contributes substantially to inter-individual variability in postprandial glycaemic (PPG) responses that result from differences in oral processing, bolus and saliva properties (Berry et al., 2020; Read et al., 1986; Tan et al., 2016).

Variation in mastication behaviour has been shown to influence PPG responses and insulin release. Within the first 30 min of the postprandial period, differences in food oral processing behaviours (OPB) and associated bolus properties can affect subsequent enzymatic activity and glucose release (Berry et al., 2020). An early demonstration of the role of mastication on PPG was by Read et al. (1986), who showed that swallowing food without chewing led to a significant reduction in PPG. Suzuki et al. (2005) showed that among a normal glucose tolerance group, longer mastication of a mixed meal produced higher insulin and significantly lower glucose responses, than their usual mastication. The same study highlighted that among the hyperglycaemic group, thorough mastication did not potentiate early-phase insulin secretion to the same extent (Suzuki et al., 2005). Similarly, Madhu et al. (2016) compared degree of mastication and found that extended mastication reduced PPG among a normal glucose tolerance group, while little effect was observed among a hyperglycaemic group. By contrast, a longer mastication time per mouthful for a fixed carbohydrate rice meal was associated with increased PPG concentrations (Tan et al., 2016). Longer oral processing time and thorough mastication per mouthful has been proposed to stimulate early insulin release and better regulate postprandial glycaemic responses, while also increasing satiety per kcal consumed (Suzuki et al., 2005; Tan et al., 2016, Zhu et al., 2013).

The number of chews per bite has been shown to influence glycaemic response and directly affect bolus particle size and surface area (Hoebler et al., 2000). Chewing for a longer time reduces average bolus particle size, increases bolus surface area and increases saliva flow and uptake. The number of mastication cycles required to form a swallowable bolus has been shown to differ widely between different foods (Peyron et al., 2004). Oral processing of food involves fracturing foods into more and smaller bolus fragments and lubricating them with saliva to form boluses that are safe for swallowing (Björck et al., 1994; Koç et al., 2013). Bolus particle size at swallow contributes to the rate and extent of starch digestibility, such that smaller bolus particles encourage more rapid starch digestion (Björck et al., 1994; Ranawana et al., 2010a, Ranawana et al., 2010b). Increasing chewing during consumption not only leads to a bolus consisting of more and smaller particles but also stimulates saliva secretion. There is natural variation across individuals in both stimulated saliva flow rates and enzymatic activity, which in turn can influence the rate and extent of starch breakdown (Atkinson et al., 2018). In this regard, extensive chewing has the combined effect of stimulating greater saliva flow, increasing the available surface area of the bolus, facilitating greater saliva uptake by the bolus and prolonging substrate-enzyme interactions in the oral cavity (Engelen et al., 2005; Motoi et al., 2013). Doubling the chews per bite reduced glycaemic response, peak glucose and overall glycaemic index for a fixed carbohydrate rice meal (Ranawana et al., 2014). Similarly, an inverse correlation was observed between bolus particle size and glycaemic response for rice, but not for spaghetti. Extending mastication increases oro-sensory exposure time, but also has an impact on bolus properties at the point of swallow. Chewing for longer both stimulates greater saliva secretion and particle surface area, and affords a longer time-period for saliva uptake by the food bolus. Differences in the particle size and total surface area of the bolus at swallow can influence digestive kinetics and post-prandial metabolic and endocrine responses to the ingested nutrients. Several studies have demonstrated how structural transformations that occur during extended mastication over a longer oro-sensory time are associated with increased PPG and insulin release (Björck et al., 1994; Blanquet-Diot et al., 2021). A comparison of particle size and glucose responses showed a positive relationship between higher percentage of smaller bolus particles and higher glycaemic responses after 30-min. This suggests that the degree of habitual mastication and bolus particle size at swallow may influence both the magnitude and pattern of an individual's glycaemic response.

The extent to which oral breakdown can influence PPG is moderated by the structure and mechanical properties of foods. Salivary amylase penetration into the bolus has been shown to vary when comparing breads differing in structure and density. Enzymatic activity was higher in the bolus of industrial bread compared to artisanal bread and whole-meal bread, despite chewing durations being similar (Joubert et al., 2017). This creates the potential to attenuate PPG by modifying food structure and texture and through this reduce the penetration of salivary amylase. Mastication can reduce the viscosity of starchy foods through the rapid action of salivary amylase, though the rate and extent of starch hydrolysis depends on the initial food structure (Hoebler et al., 1998). The mixing and interaction of starch and amylase continues as the food bolus is transported through the oesophagus to the stomach. As food arrives in the stomach, it generally has a pH of between 5 and 6, before sufficient acid is secreted to drop the pH to approximately 3, strongly reducing the enzymatic activity of salivary amylase and ending the oral phase of digestion (Bornhorst, 2017; Fried et al., 1987). This process of bolus particle breakdown, amylase mixing and initial starch hydrolysis can take approximately 25–30 min, and variations in this early phase of starch digestion are likely to contribute to temporal differences in PPG.

Oral processing behaviours are modifiable, and extensive research to date has demonstrated the influence of a food's physical and mechanical properties on oral processing behaviours (Aguayo-Mendoza et al., 2019; Forde et al., 2017; McCrickerd et al., 2017), bolus properties and saliva uptake (Pereira et al., 2006). The extent to which oral breakdown can influence PPG can be moderated by food structure and texture. Using variations in texture for similar foods (e.g. rice grains vs. rice cakes) to influence postprandial glucose responses is appealing, as individuals tend to naturally adjust their oral processing behaviours in response to the textures (de Wijk et al., 2008). Despite the appeal of the approach, few studies have investigated the impact of food texture modifications on bolus properties and their impact on PPG responses. Thus, little is known about how food texture manipulations can be used to influence oral processing behaviours and bolus properties which in return might modulate PPG responses.

The impact of oral processing behaviour on PPG is the culmination of bolus surface area, quantity and uptake of salivary enzymes, and the time period of their interaction during mastication. The current study tested whether consuming an equivalent carbohydrate load as a hard/slow test meal (rice cake) compared to a soft/fast test meal (white rice) influenced oral processing behaviour, bolus properties, and the subsequent postprandial glucose response.

## 2 Materials and methods

### 2.1 Participant characteristics

A minimum sample size of 15 participants was needed to observe a difference between food textures and oral processing behaviours with a mean effect size of 1.17 (Cohen's d_z_) at 80% power, 5% alpha level and 0.5 correlation between measures (Ranawana et al., 2010a, Ranawana et al., 2010b). To account for the differential impact of food textures on oral processing, saliva flow rate, and bolus properties, and estimated attrition rates, a sample of 40 male participants (26.5 ± 4.4 years) of Chinese ethnicity were recruited for the study.

Chinese male participants were recruited to reduce gender and ethic variations in metabolic outcomes (N = 40). All participants were of normal weight (BMI 18–25 kg/m^2^), blood pressure (≤140/90 mmHg) and fasting blood glucose (≤6 mmol/L), with healthy dentition and the ability to bite, chew and swallow normally, without history of chronic medical illness (i.e. diabetes), long-term medication or painkiller use, and no reported food allergies or intolerances to the test foods.

The study was approved by the National Healthcare Group Domain Specific Review Board (NHG DSRB, Reference Number: 2018/01091), Singapore. All participants provided written informed consent and were financially compensated for their time. The study was pre-registered at Clinical Trial registry: NCT04683432.

### 2.2 Experimental overview

The study was a randomised cross-over, within-subjects, non-blinded design. Participants attended a screening session followed by two tests sessions separated by at least 5 days at the Clinical Nutrition Research Centre, in the Yong Loo Lin School of Medicine at the National University of Singapore. Participants were overnight fasted before each test meal and completed anthropometric, blood pressure, fasting blood glucose and saliva measures during the initial screening session. During the two test meal sessions, participants were asked to consume a fixed portion of a test meal (white rice: 106.3 g (221 kcal) or rice cake: 115.9 g (219 kcal)) while being video-recorded. Oral processing behaviours were derived from post-hoc behavioural annotation of meal videos (described in section 2.4). Before and after each test meal, participants completed a series of appetite ratings to determine satiety responses and sensory ratings for each test meal. Blood samples were obtained using the finger prick method for 120 min post-meal (section 2.5). Following the final blood draw, participants were provided with a sample of each test meal for bolus collection, and saliva and bolus samples were characterized post-hoc (described in sections 2.7, 2.8).

### 2.3 Test meals and oral processing measures

White rice (Double FP brand, NTUC Fairprice Co-operative Ltd, Singapore) and Korean rice cake (Sungji brand, NTUC Fairprice Co-operative Ltd, Singapore) were selected as test meals and served in portions consisting of 50 g available carbohydrate. This amounted to a cooked weight of 106.3 g (221 kcal) of white rice and 115.9 g (219 kcal) of rice cake. White rice was cooked in a 1:1 ratio with water using a rice cooker (Toyomi RC 515). Rice cake was prepared by boiling in water for 3 min, drained, cooled to room temperature (22 °C) and served in whole pieces in its original 10 g tubular-shaped form. Both test meals were served on a plate with a fork. Participants arrived at the test centre at 9am in a fasted state and asked to ‘eat in their normal way’ and to consume the full portion of each meal. To compare oral processing behaviours for each test meal, recordings of each meal consumption were coded using an annotation software (ELAN 5.8, Max Planck Institute for Psycholinguistics, The Language Archive, Nijmegen, Netherlands) with a behavioural coding approach described previously (Forde et al., 2013, 2017). The frequencies of key ‘point’ events (bites, chews and swallows) and duration of a single ‘continuous’ event (time of food in mouth) were recorded and the microstructural eating patterns, such as *bite size* (g/bite), *eating rate* (g/min), *chews* per *bite*, *chews* per *gram* and *oral exposure time* (s) were derived.

### 2.4 Blood glucose measures

Blood samples were collected using the finger-prick method (Abbott SF Single Use Lancing Device, IL, USA) with measures taken pre-meal at baseline (0 min), and after completion of the test meal at 5, 10, 15, 30, 45, 60, 90 and 120 min. Blood glucose concentration at each time points was measured with a glucose dehydrogenase assay (HemoCue 201 RT, Sweden).

### 2.5 Appetite and sensory ratings

Participants rated their *hunger*, *fullness*, *prospective intake* and *desire to eat,* on a horizontal visual analogue scale (VAS) at baseline (pre-meal) and at nine 15 min intervals after meal consumption. After the last rating (120 min), participants were provided free access to an *ad-libitum* portion of snacks and intake was measured as the difference in snacks before and after consumption.

To compare the sensory properties of each test meal, participants rated each meal using a series of pre-defined sensory attributes including: ‘*firmness’, ‘chewiness’, ‘stickiness’, ‘springiness’, ‘sweetness’, ‘saltiness’, ‘pleasantness/liking’* on a VAS. All VAS ranged from 0 to 100 and were anchored with “Not at all” (0) and “Extremely” (100). All appetite and sensory ratings were collected using Compusense Cloud software (Compusense Inc., Guelph, ON, Canada). The instrumental texture properties of white rice and rice cake were measured using Textural Profile Analysis (TPA) using a method described previously (Wee et al., 2018). TPA was carried out using a TA·XT plus Stable Micro Systems Texture Analyser (Stable Microsystems Ltd, Surrey, England). A spoonful of white rice (5 g, 45 Ø x 30 mm) or a unit of rice cake (5.5 g, 35 × 15 × 14 mm) were used as the sample for TPA measures. Using a flat, circular compression plate (75 mm diameter, P/75), TPA was performed at 22 ± 1 °C at a compression speed of 1 mm/s up to a strain of 30%. The test settings were fixed for white rice and rice cake. The compression was performed with 5 s waiting time between the first and the second compression. Hardness (maximum force of 1st compression), adhesiveness (negative area after 1st compression), springiness (distance of height during second compression by the first compression distance), cohesiveness (area under 2nd compression/area under 1st compression), chewiness (gumminess × springiness), resilience (area after maximum force of 1st compression/area before maximum force of 1st compression), and Young's modulus (slope of stress–strain curve during the first compression from 0 to 5% strain) were obtained.

### 2.6 Saliva measures

Participant's *stimulated* and *unstimulated saliva flow rate* (ml/min) were measured using the passive drooling method. Saliva was collected between 9 and 11 a.m. to avoid circadian variations in salivary flow rates and composition (Granger et al., 2007). For stimulated saliva flow rates, participants chewed on a piece of parafilm square (0.29 g, Parafilm M PM996) to mimic gum chewing. Unstimulated saliva was collected the same manner without the use of parafilm. Unstimulated and stimulated saliva flow rate (ml/min) were calculated as the volume of saliva collected (ml) per drooling unit time (min). Salivary *α*-amylase activity (U/ml) in unstimulated and stimulated saliva was determined using a salivary α-amylase enzymatic kit (Salimetrics Assay #1–1902, Salimetrics, LLC.), based on the principles of (saliva) enzymatic breakdown of 2-chloro-p-nitrophenol linked with maltotriose, by which the concentration of 2-chloro-p-nitrophenol produced is spectrophotometrically measured at 405 nm (Cytation 5, Winooski, VT, USA) (Engelen et al., 2005).

### 2.7 Bolus evaluation

Test meal bolus samples were evaluated for *bolus particle size*, *number of bolus particles* and *total surface area* of bolus particles at the point of swallow using methods described previously (Eberhard et al., 2012; Rodrigues et al., 2014). Participants chewed a fixed pre-weighed quantity of white rice (5 g) or rice cake (5.5 g) in a single mouthful to the point of swallowing, at which point they expectorated the bolus to a pre-weighed container. Participants rinsed their mouth with water (25 g) and expectorated the remaining food particles into the same pre-weighed container. The weight of each bolus sample was recorded and derived using the following formula: Wet bolus (g) = [Weight of rinsing water (g) + wet bolus (g) + weight of container (g)] – [weight of rinsing water (g) – weight of container (g)]. Bolus *saliva uptake* was calculated as the percentage increase in weight of the wet bolus after mastication (g), and the *rate of saliva incorporation* (SIR) was expressed as the increase in weight of the wet bolus over time (g/min). Saliva uptake (%) and SIR within each bolus were calculated to derive the volume and rate at which saliva was absorbed into the boluses.

$$Saliva\;Uptake\left(\%\right)=\frac{Weight\;of\;wet\;bolus\;\left(g\right)-Weight\;of\;food\;\left(g\right)}{Weight\;of\;food\left(g\right)}\times 100$$

$$SIR\left(g/\min\right)=\frac{Weight\;of\;wet\;bolus\;\left(g\right)-Weight\;of\;food\;\left(g\right)}{Time\;taken\;in\;mouth\;\left(min\right)}$$

Expectorated bolus samples were rinsed with 100 ml TRIS buffered saline (pH 10) to inactivate salivary amylase from further breaking down the samples, followed by separation of individual bolus particles in the petri dishes (100 mm × 15 mm). Samples were dried in an oven (Memmert MEMMUF110) at 60 °C for 3 h, cooled for 1 h and scanned (Epson Perfection 4990 photo). For bolus granulometric analysis, the scanned images were processed with Image-J, Fiji analysis (Schindelin et al., 2012) to derive the *number of particles*, *particle size* (mm^2^), and *total bolus surface area* (mm^2^) for each test food sample. For baseline comparison of the change in surface area with post-masticated boluses, granulometric analysis of pre-masticated food samples were performed. White rice (5 g) and rice cake (5.5 g) were analyzed in replicates of six per test food using the procedure described above.

### 2.8 Statistical analysis

Data from one participant was excluded from the overall analysis due to issues with the meal video recording, resulting in a final data set of 39 participants. All data were presented as mean and standard deviation (SD) or standard error of means (SEM), where appropriate. All variables were tested for normality visually and statistically using quantile-quantile plot (Q-Q plot) and Shapiro-Wilk test prior to analysis. The incremental area under the curve (iAUC) was calculated using the trapezoid rule, ignoring the area beneath the baseline, for both glucose and the four appetite rating scales (Allison et al., 1995; Brouns et al., 2005). Linear mixed model was used to test for effect of food type, time and their interaction on glucose concentration and appetite ratings separately while controlling for the baseline measurements, with a random subject effect. Food type, time and their interaction were the fixed factors while the repeated subcommand was used to model the correlation in the repeated measures over time. Significant interaction between food and time was followed with pairwise comparison between the foods at each time point. Paired samples T-test was used to explore differences in sensory ratings, oral processing, saliva and bolus parameters, glucose iAUC and ad-libitum snack intake between the two test foods. Five participants were removed from the comparative analysis of bolus saliva uptake and SIR due to negative values from incomplete bolus recovery. Independent T-test, with Satterthwaite approximation for degrees of freedom, were used to test for differences in TPA parameters between the test foods. Multiple linear regression was used to identify effect of various variables (oral exposure time, saliva uptake, salivary α-amylase activity and total surface area) on iAUC_30_ (iAUC between 0 and 30 min) for the two test foods; standardised and unstandardized regression coefficients are reported. All statistical analyses were performed using SPSS (IBM SPSS statistics, version 26), with alpha = 0.05.

## 3 Results

### 3.1 Participant characteristics

Participant age, anthropometric, blood glucose and saliva measures are summarized in Table 1 and average measures were within the normal range for anthropometry, blood pressure and glucose. The unstimulated and stimulated saliva flow rates, and unstimulated and stimulated salivary amylase activity are in line with previous reports (Engelen et al., 2005; Nascimento et al., 2012; Tarragon et al., 2018).

**Table 1:** Mean (±SD) age, anthropometric, blood glucose and saliva measures of the participants (N = 39, male).

Age (years)	26.5 (4.4)
BMI (kg/m^2^)	21.2 (1.7)
Body fat (%)	14.7 (3.9)
Systolic blood pressure (mmHg)	118.7 (8.1)
Diastolic blood pressure (mmHg)	74.0 (6.9)
Fasting blood glucose (mmol/L)	4.7 (0.5)
Unstimulated saliva flow rate (ml/min)	0.5 (0.3)
Stimulated saliva flow rate (ml/min)	1.5 (0.6)
Unstimulated α-amylase activity (U/ml)	69.4 (52.6)
Stimulated α-amylase activity (U/ml)	103.6 (59.6)

### 3.2 Sensory and instrumental texture of white rice and rice cake

Participant sensory ratings for the white rice and rice cake are summarized in Table 2 together with results from instrumental texture measurements. Rice cake was significantly higher in perceived ‘*stickiness’* (*p* < 0.001)*, ‘springiness’* (*p* < 0.001)*,* and *‘chewiness’* (*p* < 0.001) than white rice. Instrumental *‘adhesiveness’* (*p* = 0.003)*, ‘chewiness’* (*p* < 0.001)*, ‘cohesiveness’* (*p* = 0.001)*, ‘resilience’* (*p* = 0.001) and *‘Young's modulus'* (*p* = 0.017) were significantly higher for rice cake than white rice. Both test foods were perceived as similar in firmness (*p* = 0.213) while TPA showed that rice cake was twice as hard as white rice. There was no significant difference in liking between test foods (*p* = 0.077).

**Table 2:** Subjective sensory ratings (N = 39) and instrumental texture profile analysis of white rice and rice cake.

Perceived sensory ratings	White rice	Rice cake	*p*-value
Firmness (mm)	67.9 (3.1)	62.1 (3.8)	0.213
Stickiness (mm)	40.7 (4.7)	80.4 (2.1)	< 0.001 *
Springiness (mm)	29.0 (3.8)	75.2 (2.9)	< 0.001 *
Chewiness (mm)	46.9 (4.5)	86.3 (2.2)	< 0.001 *
Sweetness (mm)	27.1 (3.3)	30.2 (4.0)	0.491
Saltiness (mm)	9.2 (2.0)	22.8 (3.6)	< 0.001 *
Pleasantness/Liking (mm)	32.7 (3.5)	39.0 (3.5)	0.077
**Textural profile analysis**	**White rice**	**Rice cake**	***p*-value**
Hardness (N)	2.83 (0.22)	6.78 (0.17)	< 0.001 *
Adhesiveness (N*s)	0.06 (0.03)	1.94 (0.49)	0.003 *
Springiness (-)	0.55 (0.16)	0.92 (0.01)	0.138
Chewiness (-)	39 (16)	566 (13)	< 0.001 *
Cohesiveness (-)	0.23 (0.02)	0.89 (0.00)	0.001 *
Resilience (-)	0.14 (0.02)	0.57 (0.00)	0.001 *
Young's modulus (Pa)	515 (327)	2422 (128)	0.017 *

Values are presented as mean (SEM). Sensory ratings: paired samples T-test; TPA: independent samples T-test, with Satterthwaite approximation for degrees of freedom. * Significant difference at *p* < 0.05 between test foods.

### 3.3 Oral processing, bolus and Saliva characteristics of white rice and rice cake

**Fig. 1:**
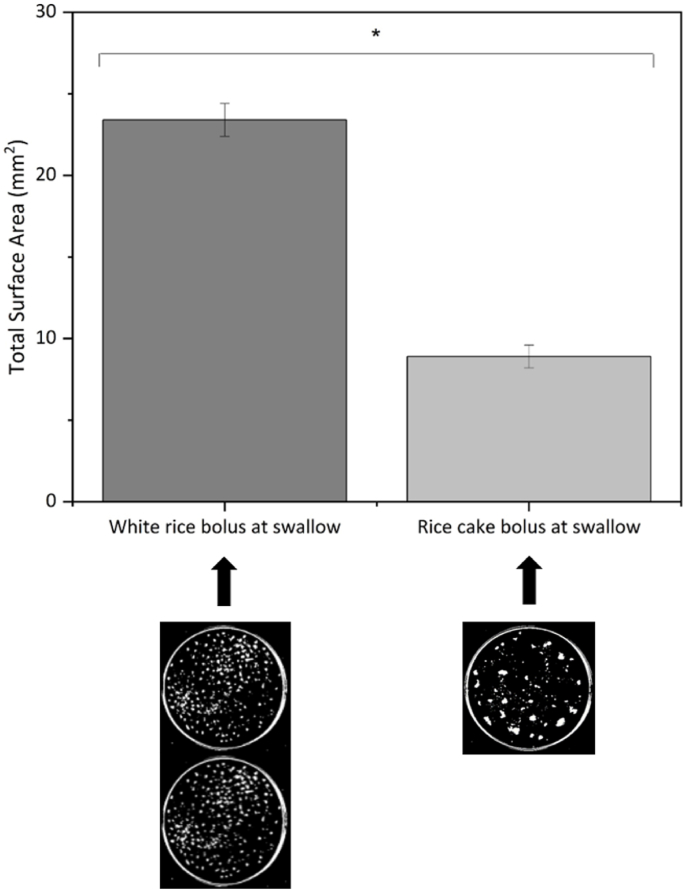
Total surface area of white rice and rice cake at the moment of swallow with accompanying images showing expectorated bolus. *indicates significant difference in total surface area of boluses at swallow between test foods, p < 0.05.

The oral processing, bolus and saliva characteristics for each test meal are summarized in Table 3. Rice cake was consumed 11% faster (*p* = 0.033) than white rice, eaten with 71% larger bite size (*p* < 0.001) and 20 additional chews per bite compared to white rice (*p* < 0.001). There was no significant difference in oro-sensory exposure time (s) between the two test meals. The white rice bolus particles were smaller (*p* < 0.001) and had a larger total surface area at swallow (*p* < 0.001) compared to the rice cake (Fig. 1). Saliva uptake (*p* = 0.038) and SIR in the bolus (*p* = 0.034) were significantly higher for white rice compared to rice cake.

**Table 3:** Differences in the oral processing, bolus and saliva properties between white rice and rice cake (N = 39).

Oral processing behaviours (meal)	White rice	Rice cake	*p*-value
Bite size (g/bite)	6.2 (0.3)	10.6 (0.4)	< 0.001 *
Chew rate (chews/s)	1.2 (0.0)	1.4 (0.0)	< 0.001 *
Chews per bite (no.)	30.3 (2.3)	49.2 (3.3)	< 0.001 *
Chews per gram (no.)	5.0 (0.3)	4.8 (0.3)	0.378
Oral exposure time (s)	424 (24)	412 (22)	0.522
Eating rate (g/min)	17.1 (1.1)	19.0 (1.1)	0.033 *
**Bolus and saliva properties**	**White rice**	**Rice cake**	***p*-value**
Number of bolus particles (−)	1125 (69)	303 (29)	< 0.001 *
Total surface area (mm^2^)	2339 (103)	894 (72)	< 0.001 *
Bolus particle size (mm^2^)	2.2 (0.1)	3.4 (0.3)	< 0.001 *
Saliva uptake (%)	58.0 (6.1)	46.8 (5.4)	0.038 *
Saliva incorporation rate (SIR) (g/min)	5.4 (0.6)	4.3 (0.4)	0.034 *

Values are presented as mean (SEM). Paired samples T-test were performed to determine * significant differences at *p* < 0.05 between test foods.

### 3.4 Postprandial glucose responses to white rice and rice cake

**Fig. 2:**
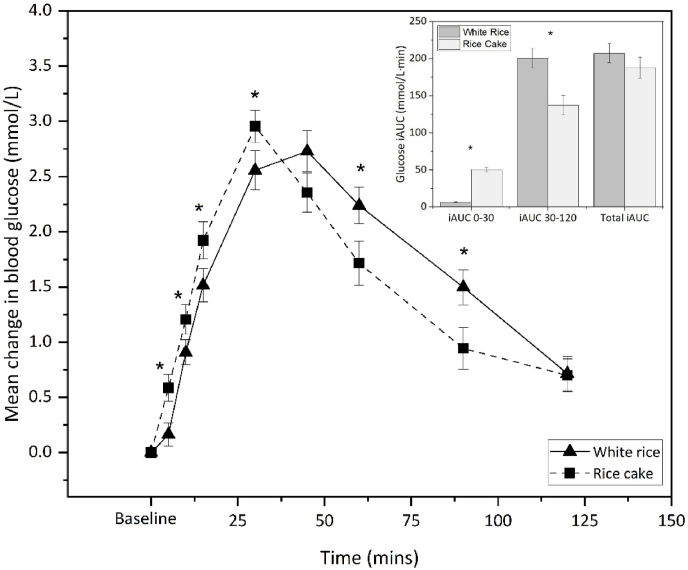
Mean change in baseline-corrected blood glucose concentration following white rice and rice cake (*N* = 39). Error bars indicate standard errors. The insert plot summarises glucose iAUC at 0–30 min, 30–120 min and 0–120 min (total iAUC). * indicates significant difference in mean iAUC between white rice and rice cake (*p* < 0.05).

The average post-prandial glucose responses for each test food are shown in Fig. 2. There was a significant interaction between type of food and time (glucose*time, *p* = 0.001) with a significantly higher postprandial glucose for rice cake than white rice (*p* = 0.028) in the first 30-mins post-consumption **(**Fig. 2**)**. The glucose iAUC was higher for rice cake during the first 30 min (iAUC_30_, *p* = 0.010), whereas glucose area under the curve was significantly higher for white rice after 30 min (iAUC_30-120_, *p* = 0.031). Despite these temporal differences in glucose concentration, the overall postprandial glucose iAUC did not differ significantly between white rice and rice cake (*p* = 0.200).

### 3.5 Impact of oral processing, bolus properties and Saliva on glucose response for white rice and rice cake

Table 4 summarises the results of the regression analyses to compare the relative influence of the different determinants of glucose iAUC_30_ for white rice and rice cake. The duration of oro-sensory exposure time was positively associated with glucose iAUC_30_ for both test meals. Longer oro-sensory exposure time was correlated with greater number of chews per bite for white rice (r_s_ = 0.727, *p* < 0.001, data not shown) and rice cake (r_s_ = 0.576, *p* < 0.001, data not shown). Food oral exposure time was inversely correlated with eating rate, where a longer oro-sensory exposure time was associated with a slower eating rate for both white rice (r_s_ = −0.998, *p* < 0.001, data not shown) and rice cake (r_s_ = −0.999, *p* < 0.001, data not shown). The saliva uptake in the bolus was positively associated with an increased glucose iAUC_30_, though this relationship was only significant for the rice cake (*p* = 0.008) and not for white rice (*p* = 0.541). Salivary α-amylase activity and the total surface area of bolus were not significant contributors to glucose iAUC_30_ after consuming white rice and rice cake (*p* > 0.05).

**Table 4:** Standardised and unstandardized regression coefficients for the relationships between oral processing, bolus and saliva properties and glucose iAUC_30_ for white rice and rice cake.

	White rice glucose iAUC_30_				Rice cake glucose iAUC_30_			
Parameters	*B* (95% CI)	SE	β	*p*-value	*B* (95% CI)	SE	β	*p*-value
**Oral exposure time (s)**	0.05 (0.01, 0.09)	0.02	0.39	0.029*	0.08 (0.03, 0.12)	0.02	0.48	0.003*
**Saliva uptake (%)**	0.06 (−0.14, 0.26)	0.10	0.11	0.541	0.26 (0.07, 0.45)	0.09	0.40	0.008*
**α-amylase activity (U/ml)**	0.02 (−0.10, 0.13)	0.06	0.06	0.743	0.00 (−0.10, 0.10)	0.05	0.00	0.975
**Total surface area (mm**^**2**^**)**	−0.01 (−0.02, 0.01)	0.01	−0.21	0.267	−0.01 (−0.02, 0.01)	0.01	−0.16	0.288

*B* = Regression coefficient. SE = Standard error. β = Standardised regression coefficients. R^2^ (white rice) = 0.181. R^2^ (rice cake) = 0.434. Multiple linear regression, N = 34, due to incomplete dataset. * Significant difference at *p* < 0.05.

### 3.6 Comparison of satiety and post-meal snack intake following consumption of white rice and rice cake

**Fig. 3:**
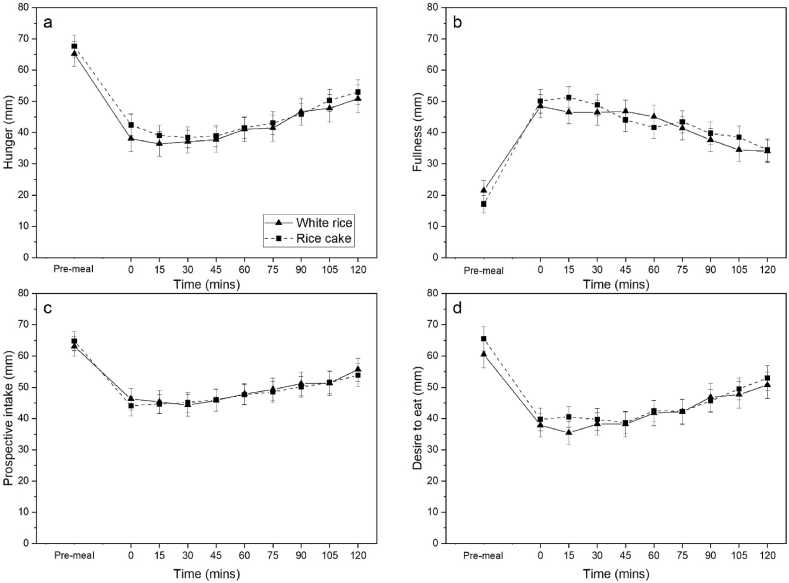
a-d. Mean appetite ratings (a. hunger; b. fullness; c. prospective intake; d. desire to eat) pre-meal to 120 min following consumption of white rice and rice cake. N = 39. Error bars are presented as SEM. There were no significant interactions between treatment and time while controlling for pre-meal appetite ratings (*p* > 0.05).

Fig. 3a–d summarises the average change in participant subjective ratings for *hunger*, *fullness*, *prospective intake* and *desire to eat* for 120 min after each test meal. There were no significant differences in subjective *hunger* (*p* = 0.926), *fullness* (*p* = 0.638), *prospective hunger* (*p* = 0.977) and *desire to eat* (*p* = 0.840) following consumption of the test meals. Food texture based differences in OPB and eating rate did not significantly influence overall post-meal satiety (total iAUC). Post-meal *ad-libitum* snack intake did not differ between white rice and rice cake meals (*p* = 0.956), with participants consuming an average of 405.2 (±34.8) kcal of snacks 2 h after the consumption of white rice, and 404.1 (±34.8) kcal of snacks 2 h after consumption of the rice cake meal.

## 4 Discussion

Using a ‘free eating’ paradigm, food texture differences significantly influenced food oral processing behaviours, bolus particle size and saliva uptake. The differences in OPB produced by food texture changes of a fixed carbohydrate meal led to temporal differences in glucose release, that were correlated with meal bolus surface area and saliva uptake. Despite temporal differences in PPG between test meals, there was no overall significant difference in postprandial glucose iAUC or satiety responses between the hard/chewy rice cake, and the soft/less chewy white rice.

Differences in test meal texture resulted in large and consistent differences in participant OPB when consuming white rice and rice cake meals. Participants consumed each meal in their normal way, and the resultant differences in oral processing show the potential for harder, chewier, and stiffer foods (i.e. rice cake) to naturally extend the number of chews per bite when compared to softer and less chewy foods (i.e. white rice). Previous research has shown correlations between instrumental texture properties of food and oral processing behaviours, and found that increasing *hardness* and *elasticity* resulted in an increased number of chews per bite and longer oro-sensory exposure time (Bolhuis and Forde, 2020; Foster et al., 2006; Wee et al., 2018). Our previous findings demonstrated that white rice (19.9 g/min) was eaten at a faster rate than rice cake (16.8 g/min) (Forde et al., 2017), which was confirmed in other studies (Iguchi et al., 2015; Shiozawa et al., 2013). This was however not replicated in the current study, as rice cake was eaten faster (19.0 g/min) compared to white rice (17.1 g/min). Despite a 63% increase in chews per bite for rice cake, there were negligible overall differences in oro-sensory exposure time. This is likely due to a unit bias, wherein the rice cake was consumed with a larger bite size due to their fixed unit size, which led most participants to consume rice cake pieces in a single bite.

The current study confirms that oral processing can influence PPG excursions through a combination of increased bolus surface area, increased bolus saliva uptake, and an extended duration for this interaction during mastication. Bolus analysis showed that white rice bolus particles were smaller, with a greater surface area and higher saliva uptake compared to the rice cake bolus. A larger bolus surface area supports greater saliva uptake, by increasing the available surface for saliva coating the bolus particles (Liu et al., 2017). This confirms previous reports that bolus particle size, rather than the number of mastication cycles, has a stronger influence on bolus saliva uptake (Motoi et al., 2013; Tournier et al., 2014). The impact of bolus particle size on starch hydrolysis within the first 30-min post-meal has been shown in a number of studies, where smaller bolus particle sizes support faster carbohydrate release and subsequently affect overall glucose release (Ranawana et al., 2011, 2014). In addition to bolus particle size and surface area, the current study highlights the importance of eating rate in contributing to overall PPG as the duration of oro-sensory exposure was also a significant predictor of PPG. Food structures that require minimal oral processing, and can be consumed quickly, may attenuate early-phase PPG by reducing the extent of salivary amylase activity on the available starch.

The consumption of rice cake led to earlier glucose response which is likely due to a higher number of chews as compared to white rice consumption. Previous studies have utilized a fixed chewing regime and shown associations between extended mastication and higher postprandial glucose and insulin responses, where the potentiation of early-phase insulin secretion aids in the regulation of postprandial glucose excursions (Lasschuijt, et al., 2020; Read et al., 1986; Suzuki et al., 2005; Zhu et al., 2013). However, despite large differences in meal texture and OPB, the current study showed no significant difference in the PPG iAUC between rice cake and white rice. The rice cake had more rapid temporal increases in blood glucose in the first 30-min post-meal, which we attribute to differences in the available starch in the pulverized glutinous rice. Rice cakes are manufactured using wet milled rice flour, whereby whole rice grains are pulverized with the endosperm cell wall structure broken down during processing. This may have resulted a greater availability of starch for digestion during the oral phase of consumption for rice cakes, compared to whole rice grains (Ren and Shin, 2013). These differences in starch structure and accessibility are acknowledged as a possible limitation for the PPG comparisons in the current study. Participants were encouraged to eat in their normal way which enabled an observational comparison of natural variations in OPB between the two test meals that differed in textures, which provided an ecological validity to the differences in PPG. While these results reflect habitual eating behaviour, the corresponding PPG results were also likely influenced by the type of available starch in the test foods, and the impact of observed differences in OPB may have been confounded by differences in the availability of substrate between the test meals, and the aforementioned unit size differences between the white rice and rice cake (Eck et al., 2019).

The current study demonstrated that variations in salivary α-amylase activity were not associated with observed differences in glucose responses for each test meal. Previous findings suggest that individuals with high salivary α-amylase activity have a lower PPG due to higher early insulin release, compared to those with lower α-amylase activity (Barling et al., 2016; Mandel and Breslin, 2012). Our finding is in line with several subsequent studies that did not replicate the relationship between α-amylase activity and blood glucose responses (Goh et al., 2021; Lasschuijt et al., 2020; Tan et al., 2016). Findings from the current trial support a greater role for bolus particle size, surface area and oro-sensory exposure time and the related extent of saliva uptake in moderating blood glucose concentrations.

Extensive chewing has previously been associated with increased satiety on kilocalorie for kilocalorie basis, such that the same quantity of food imparts a stronger satiety response when chewed for longer (Miquel-Kergoat et al., 2015). Findings from our recent study showed that when participants consumed a fixed portion at a slower eating speed during a mixed meal tolerance test, they had higher post-meal satiety compared to those eating the same meal at a faster rate (Goh et al., 2021). The current study replicated this with contrasting textured foods but did not show a significant difference in post-meal satiety between the two equi-caloric test meals. The lack of a clear difference in post-meal satiety may have been due to the lower energy served during the fixed carbohydrate meal, as participants reported being moderately full at the end of each test meal, which may not have attenuated observable differences in post-meal satiety. Previous findings have shown that modifications to a food's texture influence OPB and post-meal hunger and desire to eat (McCrickerd et al., 2017; Zhu and Hollis, 2014a, 2014b; Zhu et al., 2013). Having a longer food oral exposure time in mouth was associated with higher circulating levels of the satiety hormones PYY and GLP-1, which support increased feelings of fullness over time (Angelopoulos et al., 2014; Kokkinos et al., 2010; Lasschuijt et al., 2020). Although not confirmed in the current study, evidence suggests that texture manipulations can influence post-meal satiety and later food intake by increasing chews per bite and extending the oro-sensory exposure time during consumption (Stribiţcaia et al., 2020).

Using food texture to direct OPB creates new opportunities to regulate PPG by moderating the interaction of bolus surface area and saliva uptake during the oral phase of digestion. Large changes in food texture can have a sustained impact on food oral processing and eating rate, and there are a number of approaches to adapt food textures to influence how a food is consumed (Bolhuis and Forde, 2020). In addition to large changes to the mechanical properties of food, it is also possible to include changes at the micro-structural level to alter the rate and extent of starch digestion. For example, proteins and indigestible polysaccharides have been added to starchy foods to form semi-solid structures that extend food oral exposure time while maintaining similar sensory properties (Campbell et al., 2017). To reduce the rate of starch digestion, resistant starch can be added to carbohydrate-rich food formulation to reduce starch hydrolysis without altering the taste and appearance of foods (Fuentes-Zaragoza et al., 2010). A recent study added denatured pea protein to a noodle formulation to reduce the degree of starch gelatinization during cooking and the subsequent available carbohydrate for digestion. This led to a significant reduction of *in-vitro* glucose release (Wee et al., 2019), without any change to the noodle sensory properites. The application of micro-structural changes alongside food texture modification creates new opportunities to design foods that can moderate PPG and insulin release, and support healthier metabolic responses to food intake. These approaches are unique in that they consider the composition of a food and the habitual oral processing of the eater, to better align a person's eating behaviours with their subsequent metabolic response to an ingested nutrient.

## 5 Conclusion

The current study demonstrates that texture-differences in equivalent carbohydrate load naturally, and significantly, influenced food OPB, bolus particle size and surface area, and saliva uptake. The differences in OPB produced by textural differences of a fixed carbohydrate meal led to temporal differences in glucose release, that were strongly associated with oro-sensory exposure time and saliva uptake, though not observable for salivary α-amylase concentration. While these changes produced significant temporal differences to PPG, there was no difference in the overall PPG between the test foods. Nonetheless, food oral processing remains as an important factor in PPG, and future research should explore the potential for texture-based changes to oral processing to enhance glucose metabolism and apply sensory manipulations to food texture to support the maintenance of euglycemia.

## Funding

Supported by the Singapore 10.13039/501100012415Biomedical Research Council (Grant no. H18/01/a0/E11) Food Structure Engineering for Nutrition and Health (PI: C.G. Forde).

## Ethics

The study was approved by the National Healthcare Group Domain Specific Review Board (NHG DSRB, Reference Number: 2018/01091), Singapore.

## Clinical Trial registry

NCT04683432.

## CRediT authorship contribution statement

**J.Y.M. Choy:** Formal analysis, Data curation, Writing – original draft, Writing – review & editing, Visualization. **A.T. Goh:** Methodology, Formal analysis, Investigation, Resources, Data curation, Writing – original draft, Writing – review & editing, Project administration. **G. Chatonidi:** Formal analysis, Investigation, Resources, Writing – review & editing, Project administration. **S. Ponnalagu:** Formal analysis, Writing – review & editing. **S.M.M. Wee:** Methodology, Writing – review & editing. **M. Stieger:** Validation, Writing – review & editing. **C.G. Forde:** Conceptualization, Methodology, Formal analysis, Validation, Writing – original draft, Writing – review & editing, Supervision, Project administration, Funding acquisition.

## Declaration of competing interest

The authors declare that they have no known competing financial interests or personal relationships that could have appeared to influence the work reported in this paper.
